# Event-Based Eccentric Motion Detection Exploiting Time Difference Encoding

**DOI:** 10.3389/fnins.2020.00451

**Published:** 2020-05-08

**Authors:** Giulia D'Angelo, Ella Janotte, Thorben Schoepe, James O'Keeffe, Moritz B. Milde, Elisabetta Chicca, Chiara Bartolozzi

**Affiliations:** ^1^Event Driven Perception for Robotics, Italian Institute of Technology, iCub Facility, Genoa, Italy; ^2^Faculty of Technology and Center of Cognitive Interaction Technology (CITEC), Bielefeld University, Bielefeld, Germany; ^3^Biosciences Institute, Newcastle University, Newcastle upon Tyne, United Kingdom; ^4^International Centre for Neuromorphic Systems, The MARCS Institute, Western Sydney University, Sydney, NSW, Australia

**Keywords:** attentional selectivity, motion detection, eccentric down-sampling, spiking elementary motion detection, bio-inspired visual system, humanoid robotics, event driven

## Abstract

Attentional selectivity tends to follow events considered as interesting stimuli. Indeed, the motion of visual stimuli present in the environment attract our attention and allow us to react and interact with our surroundings. Extracting relevant motion information from the environment presents a challenge with regards to the high information content of the visual input. In this work we propose a novel integration between an eccentric down-sampling of the visual field, taking inspiration from the varying size of receptive fields (RFs) in the mammalian retina, and the Spiking Elementary Motion Detector (sEMD) model. We characterize the system functionality with simulated data and real world data collected with bio-inspired event driven cameras, successfully implementing motion detection along the four cardinal directions and diagonally.

## 1. Introduction

Most modern robotic systems still lack the ability to effectively and autonomously interact with their environment using visual information. Key requirements to achieve this ability are efficient sensory data acquisition and intelligent data processing. Useful information about the environment (e.g., how far away an object of interest is, how big it is, whether it is moving) can be extracted from sensory data. More complex interactions, for example locating and retrieving a particular resource, require an attentive system that allows robots to isolate their target(s) within their environment as well as process complex top-down information.

There are a number of ways for autonomous robots and natural organisms alike to gather information about their surroundings. Teleceptive sensors, for example those using ultrasound or infra-red light, are common in engineered systems, and are also exploited by some natural organisms for navigation and object tracking (Nelson and MacIver, 2006; Jones and Holderied, 2007). However, a closer relationship between attention and activation in the visual cortex has been observed by Maunsell and Cook (2002), showing the importance of vision when interacting and being attentive within an environment whilst performing a task. Motion detection, in particular, represents one of the important attentional cues for facilitating agent-environment interactions (Cavanagh, 1992), and is used by natural organisms to avoid obstacles, respond quickly and coherently to an external stimulus within a scene, or to focus attention to a certain feature of a scene (Abrams and Christ, 2003). Due to its wide range of applications, motion detection has been an area of research for decades and has produced a number of different detection models, ranging from gradient-based algorithms (Lucas and Kanade, 1981; Benosman et al., 2012), over local-plane fitting (Brosch et al., 2015; Milde et al., 2015) and time-to-travel methods (Kramer, 1996) to correlation-based approaches (Horiuchi et al., 1991). Gradient-based methods utilize the relationship between the velocity and the ratio between the temporal and the spatial derivative. Hence, to determine the speed and direction of the motion, the derivation of the spatial and temporal intensity for each pixel is needed. All correlation-based models share the linear and spatio-temporal filtering of measured intensities, which are functions of time and location. The best-known correlation motion detectors are the biologically derived Hassenstein–Reichardt and the Barlow–Levick models (Hassenstein and Reichardt, 1956; Barlow and Levick, 1965). The Hassenstein–Reichardt model was derived from behavioral experiments with beetles, while the Barlow–Levick model was inspired by motion detection in the rabbit's retina. In both cases one elementary motion detection unit is selective to motion in one cardinal direction (preferred direction) and suppresses output to motion in the opposite direction (anti-preferred direction) (Barlow and Levick, 1965). The models themselves (from 1956 and 1964, respectively), are still assumed to describe motion detection in organisms such as fruit flies (Borst et al., 2010; Maisak et al., 2013; Mauss et al., 2014; Borst and Helmstaedter, 2015; Strother et al., 2017). A limitation of correlation-based detectors is that, depending on the time-constant of the filters used, the detector is only receptive to a limited range of velocities. This range can be shifted by varying the parameters but always remains limited.

Environment analysis using traditional frame-by-frame visual processing generally requires a robot to extract and evaluate huge amounts of information from the scene, much of which may be redundant, which hinders the real-time response of the robot. The computational resources required for visual processing can be significantly reduced by using bio-inspired event-based cameras (Lichtsteiner et al., 2008; Posch et al., 2011), where the change in temporal contrast triggers asynchronous events. Event-based cameras perceive only the parts of a scene which are moving relative to themselves. Thus, they are idle until they detect a change in light intensity above a relative threshold. When this happens, the pixel reacts by producing an event characterized by its time of occurrence. Address Event Representation (AER) protocol allows the asynchronous readout of active pixels while providing information on the event polarity and the pixel location. As such, the camera's output are ON-events for increments in temporal contrast and OFF-events for decrements. Optical flow, the vector representation of the relative velocity in a scene, has a wide range of uses, from navigation (Nelson and Aloimonos, 1989; Milde et al., 2015), to predicting the motion of objects (Gelbukh et al., 2014). We propose that these models can also be used to direct attention toward moving objects within a scene. Recent studies have developed event-based motion detection for optical flow estimation both relying on conventional processing architectures (Benosman et al., 2012, 2014; Gallego et al., 2018, 2019; Mitrokhin et al., 2018) and unconventional neuromorphic processing architectures (Giulioni et al., 2016; Haessig et al., 2018; Milde et al., 2018). Even though the former mechanisms, which leverage standard processing capabilities, show real-time optic flow estimation with very high accuracy, they are not suited for spiking neural networks and neuromorphic processors. This is due to the way information is represented, using real values in these algorithms. Additionally, the power consumption and computational complexity in Gallego et al. (2018, 2019) is too high for constrained robotic tasks. The neuromorphic approaches on the other hand can naturally interact with spiking networks implemented on low-power neuromorphic processing architectures as information is encoded using events.

In the last decade a number of spike-based correlation motion detectors have been introduced (Giulioni et al., 2016; Milde et al., 2018). Of particular interest to this work is the spiking elementary motion detector (sEMD) proposed by Milde et al. (2018). The sEMD encodes the time-to-travel across the visual field as a number of spikes (where time-to-travel is inversely proportional to velocity). The sEMD's functionality has been evaluated in Brian 2 simulations and on SpiNNaker using real-world data recorded with the Dynamic Vision Sensor (DVS) (Milde et al., 2018; Schoepe et al., 2019). Furthermore, the model has been implemented on a neuromorphic analog CMOS chip and tested successfully. The implementation on chip presents a low latency and low energy estimate of locally occurring motion. It further offers the advantage of a wider range of encoded speeds as compared to the Hassenstein-Reichardt model, and it can be tuned to different working ranges in sympathy with the desired output. Event-driven cameras, compared with classic frame-based cameras, dramatically reduce the computational cost in processing data, however they produce a considerable amount of output events due to ego-motion. Previous implementations of the sEMD have applied a uniform down-sampling across the camera's visual field. However, recent studies have found that motion detection performance depends strongly on the location of the stimulus on the retina, due to the non-uniform distribution of photoreceptors throughout the mammalian retina (Traschütz et al., 2012). Rod and cone density in the mammalian retina is high at the fovea, and decreases toward the periphery. The non-uniform distribution of photoreceptors in the retina has a strong role in speed discrimination, and it should be taken into account as an important factor in motion estimation. Taking inspiration from the mammalian visual system (Freeman and Simoncelli, 2011; Wurbs et al., 2013), where Receptive Fields (RFs) linearly decrease in size going from the retinal periphery toward the fovea (Harvey and Dumoulin, 2011), we propose an *eccentric*, space-variant, down-sampling as an efficient strategy to further decrease computational load without hindering performances. A good approximation of the mammalian space-variant down-sampling is the log-polar mapping, describing each point in the 2D space as logarithm of the distance from the center and angle. Given its formalized geometrical distribution, the log-polar mapping provides algorithmic simplification and computational advantages, for example for tasks such as moving a robot's cameras toward a desired vergence configuration (Panerai et al., 1995), or binocular tracking Bernardino and Santos-Victor (1999). Recently, the log-polar approach has been studied also for event-driven cameras, with the proposal of the Distribution Aware Retinal Transform (DART) (Ramesh et al., 2019). Although the log-polar representation would better suit the implementation of the eccentric down-sampling, the results in polar dimension would not be comparable with the classic down-sampling of the sEMD with Cartesian coordinates. For benchmarking purposes, in this paper we use an approximate implementation of the mammalian space-variant resolution, based on Cartesian coordinates.

In this work, we propose a novel approach to spiking elementary motion detection, exploiting the non-uniform retina model as a down-sampling of the visual field. By combining the sEMD with eccentric down-sampling, this work aims to improve the computational efficiency of the motion computation and take a step toward a bio-inspired attention model where information at the center of the field of view is of higher resolution and more heavily weighted than information at the periphery, allowing robots to exploit visual information to effectively interact with their environments in real time. The proposed architecture is suitable for simulation on neuromorphic platforms such as SpiNNaker (Furber et al., 2014), and offers the possibility to be easily implemented for recorded and live input data. To the authors' knowledge, artificial motion detectors with eccentric filtering of the visual field are a novel approach to motion detection. Link to the authors' repository containing the model and the data: https://github.com/event-driven-robotics/sEMD-iCub.

## 2. Methodology

The proposed work integrates bio-inspired eccentric down-sampling with the sEMD (Milde et al., 2018). Our aim is to further decrease the computational resources required, by filtering the number of incoming events into the visual field, while maintaining a fine resolution in the center of the visual field.

### 2.1. Eccentric Down-Sampling

**Figure 1:**
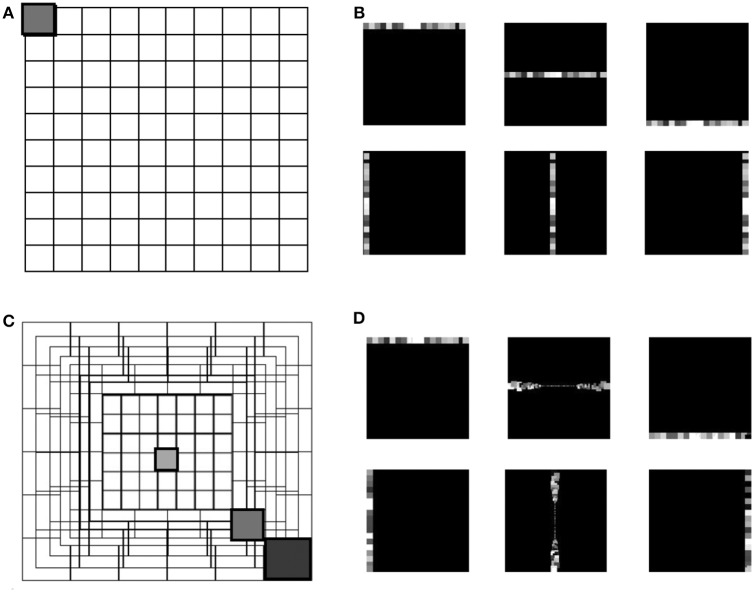
The grid in **(A)** represents the uniform down-sampling of the visual field in equal matrices of *n* by *n*. **(C)** Represents the eccentric down-sampling decreasing the size of the matrices going to the center of the visual field (fovea). This implementation does not include the blind spot present in the mammalian visual system. The three gray squares with varied hues represent three RF sizes at different eccentricities: 0, 39, 70 pixels distant from the center. The square with the same hue in both grids **(A,C)** represents a matrix with equal size in the two down-samplings. Panels **(B,D)** represent the encoding in horizontal and vertical trajectories of the uniform down-sampling **(B)** and the eccentric down-sampling **(D)**. On both top rows of **(B,D)**, an example of the RFs belonging to the first, middle and last horizontal trajectories, and on the bottom row the vertical trajectories is given. All RFs are represented with different gray-scale for the reason of visualization.

Several physiological studies have explored the mammalian retina topography such as the blind spot, fovea and eccentricities (Wässle and Riemann, 1978), showing that receptive fields are uniformly overlapped in the mammalian retina (Devries and Baylor, 1997). The proposed eccentric down-sampling approximates the two-dimensional circular retina onto a square, maintaining a quadrilateral camera resolution (Figure 1B), where each RF spatio-temporally integrates the information within its area of sensitivity. The RF size of the squared approximation decreases linearly toward the foveal region, where each RF is defined by one pixel. All RFs of the same size create a square ring around the foveal region, with each successive ring framing the previous one. The eccentric down-sampling reproduces the RF overlap between RFs of consecutive rings ensuring the robustness in response all over the retina. However, the proposed model does not include the central blind spot present in mammalian retina.

Equations (1) and (2) describe the relationship between the receptive field size (*R*^*s*^) and its distance from the foveal region, where ($R_{i}^{c}$) is the center of the top left RF of each squared ring and *i* = [1, .., *n*] is the number of squared rings over the retinal layer. The term *x* in Equation (1) represents the x axis of the camera where the origin is placed in the top left corner, *max*[**R**^*s*^] is the maximum kernel size of the outermost peripheral ring, and *d*_*fovea*_ is the total distance from the periphery to the edge of the fovea.

(1) $$\begin{array}{r} {\text{R}}^{s}\left(x\right)=-\frac{max\left[{\text{R}}^{s}\right]}{d_{fovea}}x+max\left[{\text{R}}^{s}\right] \end{array}$$

(2) $$\begin{array}{r} {\text{R}}_{i}^{c}=R_{i-1}^{c}+\frac{R_{i-1}^{c}}{2} \end{array}$$

(3) $$\begin{array}{r} {\text{M}}_{t}=M_{t-1}e^{-\frac{dt}{\tau }}+\frac{1}{R_{nf}} \end{array}$$

Each RF is a matrix of input pixels from the sensor. Every RF is modeled as a leaky integrate and fire (LIF) neuron integrating the information in space and time (Equation 3), where *M* is the membrane potential of the RF, *t* represents the temporal information of the incoming event into the RF, *dt* the difference in time with the previous event in the RF, and τ is the time constant of the exponential decay (τ = 1, 000*ms*). The membrane potential of every RF integrates incoming spikes until it reaches the threshold (*threshold* = 1), which is the same for all RFs. The contribution of each event to the increase in membrane potential of a neuron is normalized with the dimension of the RF. As the activity of the ATIS is sparse, the normalization factor (*R*_*nf*_) is expressed as a percentage of the area of the RF. Every incoming event triggers the updating of the membrane potential by calculating the temporal decay of the membrane since the last event. In addition, the membrane potential is increased by the normalization factor. This way, the response from all RFs is normalized by their occupied space over the visual field. Finally, if the threshold is reached, the neuron emits an output spike. Hence, the response from each RF coherently encodes the input information in relationship with the distance from the fovea.

### 2.2. The Spiking Elementary Motion Detector (sEMD)

**Figure 2:**
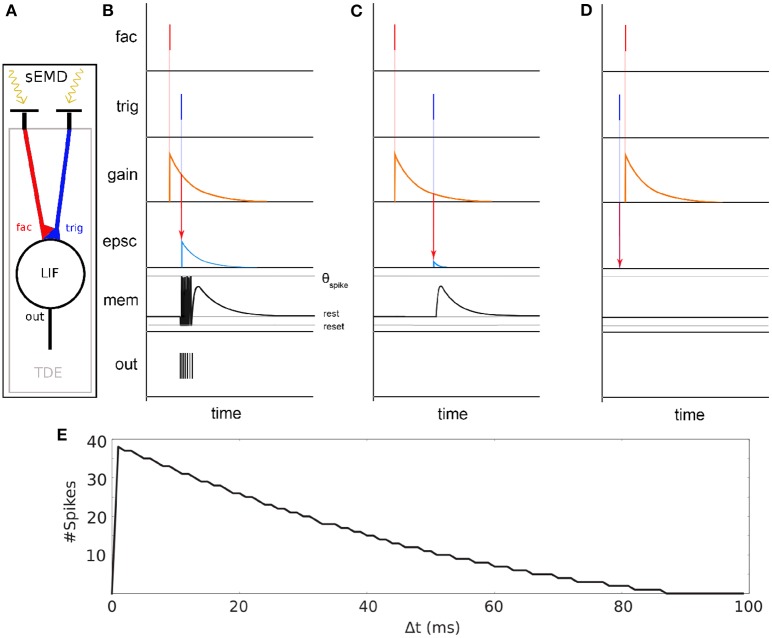
Basic principle of the sEMD (Milde et al., 2018). **(A)** The model consists of an event-based retina sending events into the Time Difference Encoder (TDE). Two adjacent RFs are connected to the facilitation synapse and the trigger synapse, respectively. **(B)** TDE computation for a small time difference Δ t between facilitation event and trigger event. An event at the facilitation synapse generates an exponentially decaying factor called gain. A trigger pulse at the trigger synapse shortly after causes an exponentially decaying Excitatory Post Synaptic Current (EPSC). The EPSC amplitude depends on the gain factor. The EPSC integrates onto the membrane potential (mem). Every time the membrane potential reaches the spiking threshold (τ_*Spike*_) an output digital pulse is produced. **(C)** Similar to **(B)** part but with high Δ t. **(D)** Similar to **(C)** but the trigger pulse arrives before facilitation pulse. No output spikes are produced for negative time differences. **(E)** TDE output spike response over time difference Δ t between facilitation event and trigger event.

The spiking Elementary Motion Detector (sEMD) depicted in Figure 2 has been designed for the purpose of encoding optic flow using event-based visual sensors (Milde et al., 2018). The use of event-based sensors is suited to perceiving motion. The edge of an object moving from the receptive field of one pixel to the adjacent one generates a spike in the two pixels with a given time difference, depending on the velocity of the edge and its distance from the pixels. The relative motion or optic flow is inversely proportional to this time-to-travel. An sEMD is composed of two pixels and a time difference encoder (TDE). The TDE encodes the time difference between two pulses into the number of output spikes produced in response to the second input pulse. The number of output spikes encodes the motion flow of objects moving in front of the two pixels.

The synapses connecting the inputs to the TDE are of two types - facilitator and trigger (see Figure 2 fac and trig). The facilitator synapse gates the activity of the TDE neuron. The trigger synapse elicits a response from the TDE neuron only if its input event occurs after the event from the facilitator synapse (compare Figures 2B,D). The output current of the trigger synapse increases the TDE neuron's membrane potential as shown in Figure 2C). The strength of the current depends on the exponentially decaying gain variable of the facilitator synapse. Therefore, the TDE not only detects the direction of motion but also encodes the velocity of the stimulus in the number of output spikes and time to first spike. The faster the stimulus propagates, the more spikes are produced by the TDE. In order to mitigate the noise present at the output of a silicon retina, a pre-processing filtering stage is used. It consist of neural spatio-temporal filters (SPTCs) used to detect correlated events. Two uniform neighborhoods, of *n* by *n* pixels, are connected to a LIF neuron each. The neurons fire once only if within a specific time, defined by their time constant, 66% of the pixels in the neighborhood produce events. The proposed implementation exploits the eccentric down-sampling (Chapter 2.1) replacing the uniform filtering stage previously used with the sEMD model by Milde et al. (2018).

### 2.3. Experiments

**Figure 3:**
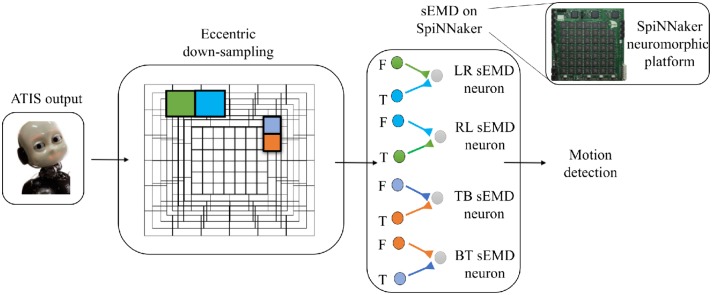
Basic scheme of the pipeline. From left to right the ATIS output is processed by the eccentric down-sampling model and sent to the sEMD model, hosted on SpiNNaker neuromorphic hardware. The sEMD model represents the layer of neurons producing spikes and encoding the motion detection. The eccentric down-sampling and the sEMD model representation show the spatio-temporal filter neurons (green, blue, violet, and orange square), the facilitator and the trigger, both synaptically connected to the sEMD neuron. Facilitators (F) and triggers (T) are shown for LR sEMD neuron, RL sEMD neuron, TB sEMD neuron, and BT sEMD neuron.

The objective of this work is to quantitatively and qualitatively characterize the output of the TDE population receiving input from the eccentricity filtering layer and to compare it with the TDE population receiving input from a uniform resolution filtering layer. This characterization aims to demonstrate the advantages of our proposed model, namely a decrease in computational load whilst maintaining the ability to estimate the velocity of moving entities within the visual field. To this purpose we characterized and compared the model using moving bars with 1D and 2D motion. In the following, we will refer to the two different implementations as “sEMD with uniform down-sampling” and “sEMD with eccentric down-sampling.” The characterization of the proposed motion detection system (Figure 3) is achieved using simulated data. Furthermore, additional experiments are undertaken using real input^1^ collected with ATIS cameras (Posch et al., 2011) mounted on the iCub robot (see Supplementary Materials for real-world data). The simulated data used in this work reproduces the activity of an event driven sensor in response to a bar moving horizontally [Left to Right (LR), Right to Left (RL)], vertically [Top to Bottom (TB), Bottom to Top (BT)] and transversely, i.e., along the diagonal of the Cartesian plane.

Firstly, we recorded the activity of the sEMD with uniform down-sampling and eccentric down-sampling model, while the speed of the input bar ranges from 0.01 to 1 px/ms, in accordance to the experiments of Giulioni et al. (2016). This ideal input allows a comparison of the two model's spike raster plots and mean population activities.

We first analyzed the selectivity of all sEMDs tuned to the same movement direction, measuring the mean firing rate (MFR) of the whole population. Given the symmetrical connectivity of the sEMD neurons along the eccentric visual field, the responses from the population of LR, RL, TB, and BT sEMD neurons are expected to be comparable, responding with a large MFR to a stimulus moving along their preferred direction and being unresponsive to a stimulus moving along their anti-preferred direction.

Further investigations focus on a single population and its response to its preferred stimulus direction (from left to right, or top to bottom), assuming transferable responses for the other directions.

A deeper understanding of the temporal response from the neurons was achieved by collecting the spike raster plots for nine speeds of the chosen range: (0.01, 0.03, 0.05, 0.07, 0.1, 0.3, 0.5, 0.7, 1 px/ms), respectively.

For each speed, we analyzed the response of each sEMD in the population, mapping its MRF onto the Cartesian space and visualizing spatial rather than temporal information. We analyzed how the Mean Firing Rate (MFR) of each sEMD changes with speed and distance from the center of the field of view. Additional experiments have been performed changing the length of the stimulus, by recruiting more sEMDs, should increase the MFR of the whole population tuned to the corresponding stimulus direction. Eventually, we analyzed the response of the model to a bar moving transversally exploring the response from the population to 2D motion. In such a case, the stimulus does not elicit the maximum response of any sEMD, rather, it elicits intermediate activity in more than one sEMD population, that need to be combined to decode the correct input direction.

### 2.4. Experimental Setup

In all experiments the model was simulated on a SpiNNaker 5 board hosting 48 ARM-chips, each with 18 cores. The SpiNNaker architecture supports highly parallelized asynchronous simulation of large spiking neural networks in almost real-time. The aspect of real-time computation is of utmost importance for the interaction of the robot with the environment. For the implementation of the SNN we chose 160 ×160 pixels as a retinal layer resolution, to limit the number of neurons to be simulated on SpiNNaker and to further minimize the impact of the residual distortion in the fringes of the camera after calibration. The output of the retinal layer serves as input to the uniformly and eccentrically down-sampled filtering layer, respectively. For the uniform down-sampling sEMD, we chose a non-overlapping neighborhood matrix size of 4 ×4 ATIS pixels to represent one RF. This filtering layer is simulated on SpiNNaker and consists of 1600 LIF neurons. It receives input from a SpikeSourceArray, containing the respective ATIS pixel spike times. The synaptic weight of the connections is 0.3. In contrast, the fovea (1 RF = 1 pixel) of the eccentric down-sampling covers 10% of the total retinal layer, and the biggest receptive field has a dimension of 10 ×10 pixels with a normalization factor of 60% (Equation 3). The population is made up of 8836 LIF neurons. The eccentric down-sampling occurs locally before the spike times of the respective receptive fields are transferred to SpiNNaker in a SpikeSourceArray. The final layer of the network consists of four sEMD populations sensitive to local motion in one cardinal directions, respectively, using sEMD neuron model included in the extra models of the pyNN library. The sEMD populations were connected to the filtering layers along the trajectories as shown in Figure 3. The combination of the output of the four populations allows the encoding of transversal stimuli. Each population shares the size of the down-sampling population. For both down-sampling approaches all sEMD neuron and synapse parameters are the same. The connectivity of the respective sEMD populations are displayed in Figure 3. The synaptic weights are 0.3 and the synaptic time-constants τ_*ex*_ and τ_*in*_ are both 20 ms. The neuron parameters amount to: a membrane capacitance of 0.25 nF, and time-constants τ_*m*_ and τ_*rf*_ of 10 ms and 1 ms, respectively. The reset, resting and threshold voltage of the neurons are defined as −85, −60, and −50 mv, respectively. To avoid a response of the sEMD-populations perpendicular to the preferred direction, in case of a bar moving their facilitator and trigger synapses receive input at the same time, the input to the facilitator synapse was delayed by 1 ms.

## 3. Results

**Figure 4:**
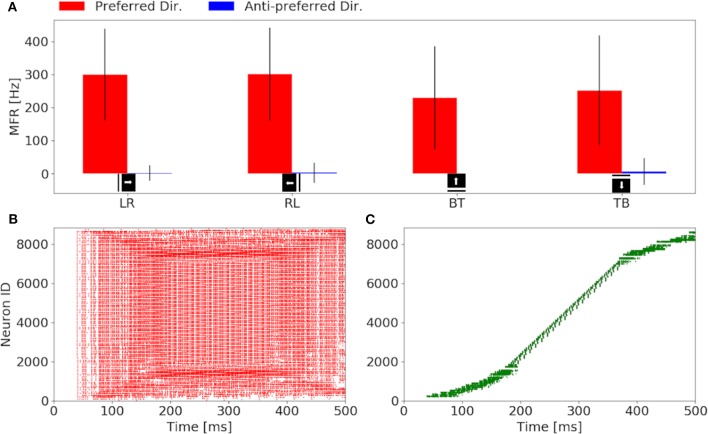
Response of the sEMDs with eccentric down-sampling to a simulated bar moving with a speed of 0.3 px/ms: **(A)** Instantaneous MFR and variance of the four sEMD-populations, each tuned to one of the four cardinal directions, to the preferred and anti-preferred stimulus. Similarly to the sEMD with uniform down-sampling, the response to the anti-preferred stimulus is negligible with respect to the response to the preferred direction stimulus. **(B)** Raster plot of the left to right (LR) population in response to a vertical bar moving from left to right. In the first 100 ms, the difference in the size of the RF can be seen, as the active neurons spike with different spike rates and the number of active neurons increases with time, when the bar moves closer to the fovea. **(C)** Raster plot of the top to bottom (TB) population in response to an horizontal bar moving from top to bottom. The sigmoidal shape arises from the geometry of the eccentric down-sampling and the neurons' indexing.

Our investigation starts with the characterization of the eccentric down-sampling sEMD's response to a simulated bar moving in the four cardinal directions with a speed of 0.3 px/ms: left to right, right to left, top to bottom and bottom to top. Figure 4 shows the response to stimuli moving in the preferred and anti-preferred directions at fixed velocity 0.3 px/ms (the middle of the regarded velocity range). In particular, Figure 4A shows the mean instantaneous firing rates of the preferred and anti-preferred direction populations. The preferred directions are colored in red and the anti-preferred directions in blue. As expected, the preferred direction population's response is significantly higher than the response of the anti-preferred direction population. Furthermore, as expected the response from all the populations to the respective preferred direction is similar in terms of instantaneous firing rate and mean firing rate, and comparable among each other, thus validating the assumption that the response to stimuli in the preferred direction is similar for all of the populations. Assuming a bar moving across the retina at a constant speed, the high variances in preferred and anti-preferred directions can be explained by the difference in receptive field sizes in our proposed model (see Figure 1). Depending on the stimulus speed, the size of the RF determines a period of time in which the stimulus moves over the RF. Thus, for the same stimulus speed, a peripheral RF takes more time to respond than one in the foveal region, leading to a different RF rings having a different sensitivity to stimulus speed. Only the RFs along the same squared ring have the same sensitivity to the same speed. If a bar is moving across the visual field at a certain speed, only neighbor RFs, that produce spikes able to trigger the TDE neurons, will detect the stimulus. Consequently, due to the varying RF sizes and varying speed sensitivities, the size of the RF relative to its neighbor affects the response of the TDE. This causes the visual field to respond non-uniformly. Figures 4B,C show examples of characteristic raster plots of the preferred direction populations, in response to a bar stimulus moving horizontally and vertically, respectively. The color-coding indicates the direction sensitivity of the population: left to right (red) and top to bottom (green). The first response to the horizontal and vertical bar movement (Figures 4B,C), is delayed by 40 ms. This is due to the stimulus taking 30 ms (speed of 0.3 px/ms) to travel over the first peripheral RF (10 ×10 px), before reaching the RF connected to the trigger. In the first 50 ms of reaction to the stimulus, the resulting spike density is rather sparse, caused by a lower response from the peripheral RFs (sensitive to higher speeds). Conversely, from 150 to 400 ms, the time where the stimulus is expected to cross the fovea, the spike density is higher because the RFs at the fovea are of a size more suited to the stimuli velocity. The impact of the proposed model is more clearly visible in response to the vertically moving stimulus (Figure 4C). The mapping from the eccentric receptive fields to the neuron IDs transforms the time sequence of a vertical bar response to a sigmoid. By contrast, the output of the sEMD with uniform down-sampling resembles the shape of stairs, with each row activated after one another, spiking with the same rate. The non-uniform size of the RFs in our proposed model is again the cause for the different spike densities produced in response to the stimulus moving at constant velocity. In this experiment the sEMDs successfully encode the direction of the bar stimulus moving across the visual field in all the four cardinal directions, showing a negligible response to the anti-preferred direction. This therefore shows that the eccentric down-sampling preserves the ability of the sEMD populations to encode optic flow of moving stimuli.

**Figure 5:**
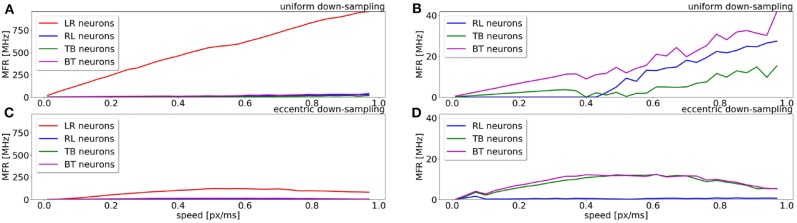
Comparison of the sEMD model with the uniform down-sampling **(A,B)** and the eccentric down-sampling **(C,D)** in response (MFR) to a left to right moving bar (simulated data). The preferred direction is displayed in red (LR), with the anti-preferred direction in blue (RL). The response for the top to bottom (TB) and bottom to top (BT) populations are displayed in green and magenta, respectively. Panels **(B,D)** are a magnification for the anti-preferred direction (right to left) and the incorrect directions (top to bottom and bottom to top) of the panels **(A,C)**. The Figure compares the behavior from the populations of the two approaches to the same stimulus and over the same range of speeds.

A comparison of the MFR for all populations of the uniform down-sampling model and the eccentric down-sampling model in response to a simulated stimulus moving from left to right at different velocities is shown in Figure 5. The color-coding remains the same as in Figures 4B,C, additionally the response of the populations selective to stimuli from right to left and bottom to top is depicted in blue and magenta, respectively. Figure 5A shows the behavior of the uniform down-sampling model, and Figure 5C depicts the behavior of the eccentric down-sampling model. Both methods show a trend of increasing MFR until target velocity reaches 0.6 px/ms. While the response from the sEMD with uniform down-sampling keeps increasing after 0.6 px/ms, the firing rate of the population with eccentric down-sampling gradually reduces as the target velocity approaches 1.0 px/ms. The same trend can also be seen for targets moving in the anti-preferred direction. Figure 5 shows that, while the sEMD response of the anti-preferred (right to left) and the incorrect directions (top to bottom and bottom to top) of the uniform down-sampling model (Figure 5B) linearly increases until 1.0 px/ms, the output firing rate of the proposed eccentric down-sampling model (Figure 5D) increases for target speeds up to 0.5 px/ms and decreases thereafter. Despite the number of sEMDs required for the proposed model (8,836 per population) being significantly higher than for the uniform down-sampling (1,600 per population) under the same setup conditions, the eccentric sEMDs' down-sampling shows an overall significant decrease in the mean output firing rate of the whole population in response to the same stimulus. Differently from frame-based systems, where the number of operations—and hence power consumption—depend on the number of filters, in event-driven spiking architectures, filters are active (and consume power) only when they receive input spikes and produce output spikes. Figure 5 shows that the proposed eccentric down-sampling model is able to differentiate between stimulus in preferred and anti-preferred directions more efficiently than a model with uniform down-sampling, without sacrificing performance. The proposed model still maintains an order of magnitude difference between MFR for stimulus in the preferred direction vs. anti-preferred direction. Although the eccentric down-sampled model does not allow for an inference of stimulus velocity to be made based on the MFR of the entire population, the same information can be extracted based on the eccentricity of the RFs with the greatest MFR.

**Figure 6:**
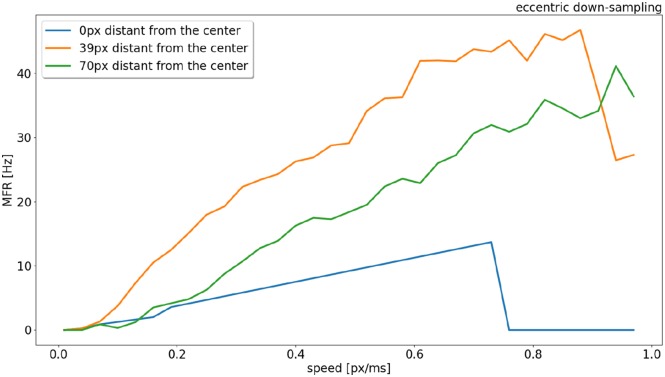
Response (MFR) to a left to right moving bar (simulated) from RFs (eccentric down-sampling) of the central horizontal line of the visual field at different eccentricities (distances from the center of the field of view). In blue, orange and green at 0, 39, and 70 pixels distant from the center, respectively (see Figure 1).

The response from sEMDs selected at different eccentricities (at 0, 39, and 70 pixels distant from the center) is examined in Figure 6 in relation to the same speed range. In the original model (Milde et al., 2018) the MFR of all three neurons would increase proportionally to the target speed. Figure 6 shows that the speed encoding for our proposed model depends on the RF size, because the integration time for each RF size corresponds to a specific range of velocities. This leads to a specific range of time-differences between two connected RFs. Each sEMD has a speed limit, which depends on its tuning, above which it will be unable to detect motion. Figure 2E shows the TDE output spikes over time difference. If a trigger event occurs before the output of the facilitation event has had time to reach the minimum threshold required, the sEMD will not fire. Due to the varying sensitivity of different RF sizes and enhanced by the 1 ms synaptic delay of the facilitator synapse, while the response from the foveal region (0 px distance) drops to zero for speeds higher than 0.7 px/ms, the response from the neuron with a middle eccentricity (39 px distance) begins to decrease dramatically at 0.9 px/ms. The response from the peripheral neuron keeps increasing until the end of the examined speed range (1.0 px/ms). A possible explanation for the relatively low MFR of the peripheral neuron is the increased number of events needed to trigger the RF and its specific sensitivity to higher speeds. Figure 6 shows how the RF size affects the behavior of the correspondent neuron, obtaining a wider operative range from the whole population. In comparison, uniform down-sampling where all the RF sizes are the same provides a comparatively limited operative range.

**Figure 7:**
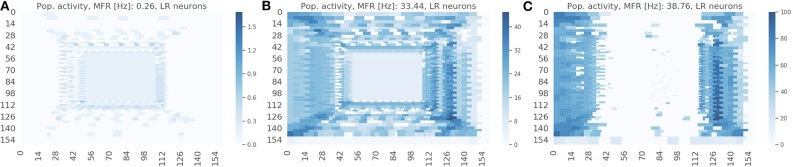
Response from the population of sEMDs with the eccentric down-sampling mapped into the cartesian space with a camera resolution of 160 ×160 pixels. The color-code heatmap represents the MFR of each RF. The stimulus was a bar moving (simulated data) from left to right with constant speed: 0.03 **(A)**, 0.3 **(B)**, 1.0 **(C)** px/ms, respectively.

The spike raster plots (Figures 4B,C) provide the temporal response from the population but they do not provide any spatial information. The visualization in Figure 7 maps the response of the sEMDs to the corresponding x and y locations for three different speeds: slow (0.03 px/ms, Figure 7A), medium (0.3 px/ms, Figure 7B) and fast (1.0 px/ms, Figure 7C). The data displayed in Figure 7B corresponds to the spike raster plot in Figure 4B. Figure 7 shows that the MFR of the whole population increases in relation to the speed: 0.26, 33.44, 38.76 Hz, respectively. The spatial visualization highlights the function of the eccentric down-sampling. As proposed by Traschütz et al. (2012), the slow speeds are detected primarily in the foveal region, where RFs have the smallest dimension and are closest to one another (Figure 7A). As the stimulus speed increases, the peripheral region starts responding from the first squared ring around the foveal region (Figure 7B) to the rings with the largest RF size for the fast speed (Figure 7C).

The response for each RF square ring is different for horizontal and vertical components (most obvious example being in Figure 7C. This is because the sEMDs in this case are only connected horizontally (as we are working with left-right motion). Therefore, at the left and right peripheries, there is a descending and ascending scale of RF sizes approaching and moving away from the foveal region, respectively. A concentrated region of diverse, overlapping connected RFs improves the likelihood of the sEMDs picking up the stimulus motion. This does not exist in the regions above and below the fovea, in which each RF will only be connected to horizontally adjacent RFs of the same size, hence the relatively low MFR in these regions.

The response on the right side of the visual field is attenuated in Figures 7B,C because the sEMDs from the last RF ring are not connected with any subsequent facilitator (although this does not cause a problem in detecting stimuli entering the scene).

**Figure 8:**
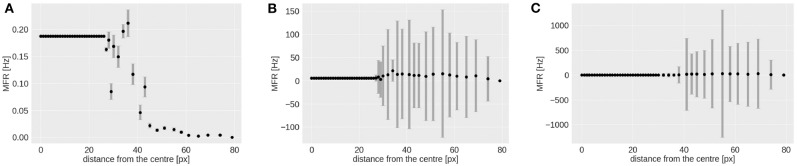
Mean and variance in MFR of RFs at different distances from the center of the visual field. The stimulus is a moving bar (simulated data) going from left to right at speeds of: 0.03 **(A)**, 0.3 **(B)**, 1.0 px/ms **(C)**.

As shown in Figure 7, the RF-ring of maximal response appears to move toward the periphery with increasing velocities. Figure 8 shows the mean and variance of the MFRs at different eccentricities for velocities 0.03, 0.3, and 1.0 px/ms, Figures 8A–C, respectively. It is clearly distinguishable, that the maximal response in MFR shifts toward the periphery with increasing velocities.

The higher variances observed at greater eccentricities (distance from the center) in Figures 8B,C, can be explained by the different RFs response from the horizontal and vertical component of the squared rings (which can be seen in Figure 7). The low MFR at 29 pixels (Figure 8A) from the center (fovea region from 0 to 28 px) can be explained by the connections between RFs of the first peripheral squared ring (about 3 ×3 px) and the fovea, where each RF has a dimension of 1 px. This sudden increase in size leads to a delay in response from the TDE receiving input to the trigger synapse from the larger receptive field.

**Figure 9:**
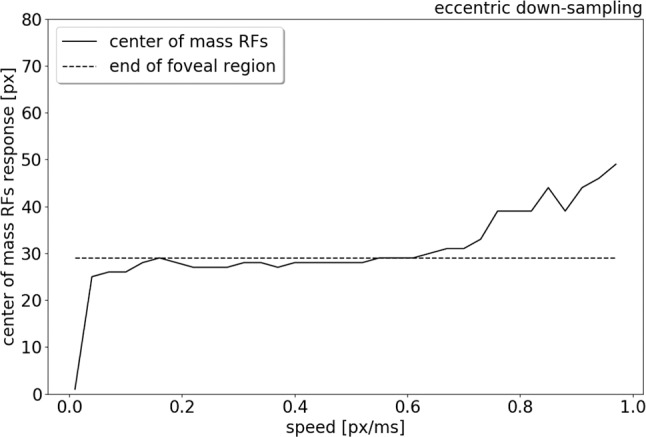
Center of mass (solid line) of the neurons response location to a left to right moving bar (simulated data), from 0.01 to 1.0 px/ms. The dash line indicates the end of the foveal region.

To compare the trend of the RFs' peak response increasing in eccentricity with increasing stimulus speed, the center of mass of the RFs response is plotted in relation to the speed range, from 0.01 to 1.0 px/ms (see Figure 9). Figure 9 shows that for low speeds (0.01–0.06 px/ms) the center of mass of the RFs' response shifts from 0 to 27 pixels (distance from the center). The center of mass then plateaus from 0.06 to 0.6 px/ms, where only the RFs of the edges of the foveal region respond to the stimulus. For higher speeds (from 0.6 to 1.0 px/ms), the eccentricity of the center of mass of RF responses starts to increase again, due to a lack of activity in the fovea. The center of mass of RF responses eventually shifts to the periphery, reaching a distance of 49 px from center.

**Figure 10:**
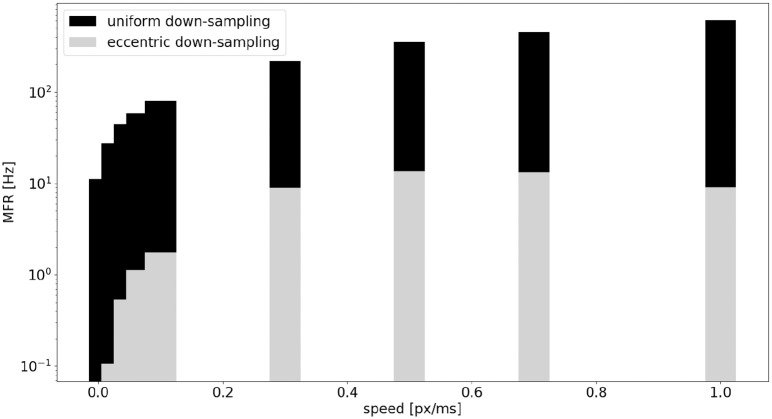
Comparison, between the sEMD model with the uniform down-sampling (1,600 neurons) and the eccentric down-sampling (8,836 neurons), of MFR from the LR sEMD neurons in response to a left to right moving bar.

A comparison of the MFR of the sEMD with uniform down-sampling and eccentric down-sampling has been explored with simulated data. Figure 10 shows the difference in response, normalized for the total number of neurons, from all populations of sEMD neurons with uniform down-sampling and eccentric down-sampling. Even though the uniform down-sampling model has fewer neurons than the eccentric down-sampling model (1,600 compared to 8,836 neurons, respectively) the MFR from the eccentric down-sampling is considerably less at each explored speed, increasing computational and power efficiency.

**Figure 11:**
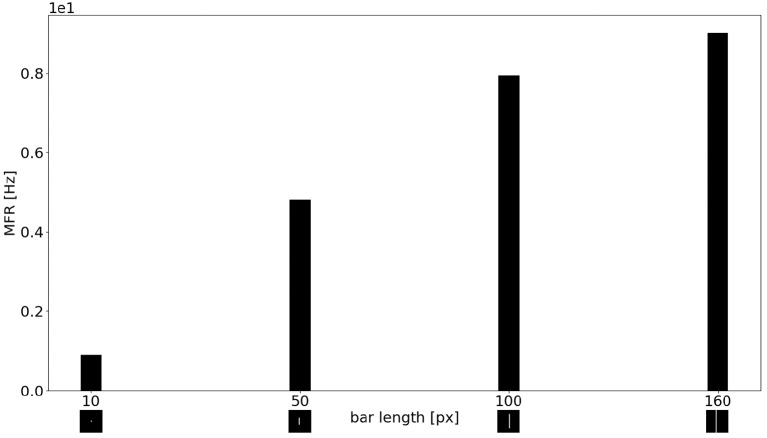
MFR response of the sEMD the LR sEMD neurons for a left to right moving bar at 0.3 px/ms with different bar lengths: 10, 50, 100, 160 pixels, respectively.

**Figure 12:**
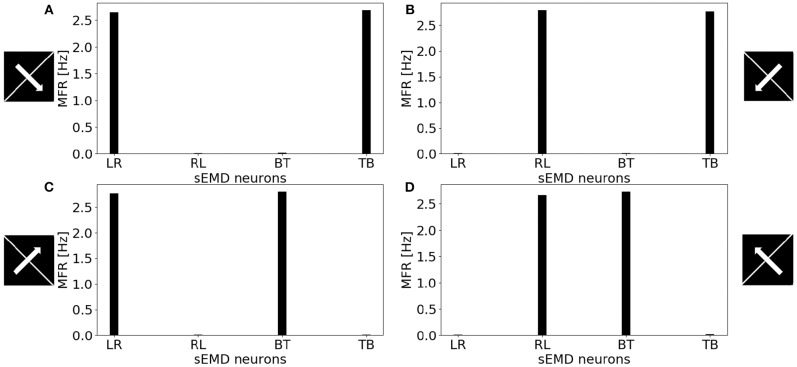
MFR response of the sEMD neurons reacting to a bar moving transversely at 0.3 px/ms. **(A)** Bar moving from the top left corner to the bottom right corner, **(B)** bar moving from the top right corner to the bottom left corner, **(C)** bar moving from the bottom left corner to the top right corner and **(D)** bar moving from the bottom right corner to the top left corner. The length of the bar covers the whole visual field.

Figure 11 shows the MFR from the population of LR sEMD neurons in response to a stimulus moving from left to right, at a medium speed of 0.3 px/ms, with bars of varying lengths: 10, 50, 100, and 160 pixels, respectively. The plot shows a positive correlation between the size of the bar and the response from the neurons sensitive to the corresponding direction. Figure 11 shows that the MFR increment decays as the length of the bar increases - most noticeable when comparing the difference in MFR between the 50 and 100 px bar, and that between the 100 and 160 px bar. This is because the bar is vertically centered in the visual field, and so longer bars cover more of the peripheral region—where each RF requires a greater number of events in order to be activated. Finally, Figure 12 shows the behavior of the population to a bar moving transversely, revealing the response of the model to 2D motion. Figure 12A shows the response to a bar moving from the top left corner to the bottom right, Figure 12B from the top right corner to the bottom left, Figure 12C from the bottom left to the top right corner and Figure 12D from the bottom right corner to the top left.

All the explored cases report a similar response from two kind of sEMD populations and a response close to zero from the other neurons. The combination of the responding sEMD neurons successfully detects the transverse motion, showing similar MFR values of the neurons that actively respond.

## 4. Discussion

The biological role of detecting temporal changes comprise two mechanisms: the detection of fast and slow movements. The first one to identify an entering stimulus into the scene and the latter one to recognize its spatial structure (Murray et al., 1983). Sudden onset of motion can attract our attention (Abrams and Christ, 2003, 2005, 2006). Hence, fast movements, speed and acceleration similarly increase our perception of a threat—making it a noticeable stimulus and grabbing our attention (Howard and Holcombe, 2010). Thus, motion detection collaborates with attentional mechanisms to react on time and interact with the surrounding.

In this paper, we have presented a novel implementation of motion detection based on the use of spiking elementary motion detectors coupled with non-uniform down-sampling inspired by the mammalian retina. The proposed model successfully detects the correct direction of an edge moving in the field of view at speeds ranging from 30 to 1,000 px/s, being suitable for the coarse motion processing of robots interacting with the environment (Giulioni et al., 2016).

With respect to the uniform down-sampling implementation presented in the original work (Milde et al., 2018), the eccentricity model significantly decreases the overall activation of each motion detector at every investigated speed. The reduced spiking activity makes this implementation more power efficient even in face of an increased number of elementary motion detectors. To achieve the same result in the uniform down sampling implementation, the size of the spatio-temporal filters should be increased, at the cost of a coarser resolution in the whole visual field and a reduced sensitivity to low velocities. The eccentricity implementation overcomes this issue maintaining the sensitivity for low and fast speed – distributed over different regions of the field of view – while significantly reducing the number of incoming events to be processed by the down-stream computational layers.

In the proposed non-uniform down sampling, the elementary motion detectors are tuned to different ranges of speed depending on their position in the field of view. The peripheral sEMDs are characterized by large receptive fields and are hence tuned to higher speeds, that progressively decreases toward the fovea. Hence, the proposed implementation encodes the speed based on the location of the active sEMD. RFs with similar size work in a similar range of speed producing redundant information, and making the decoding of the population activity robust. Moreover, thanks to the sensitivity to high speeds of the peripheral RFs, the detection of objects moving into the visual field is immediate. The sEMDs in periphery will trigger a response to a fast stimulus entering the field of view with extremely low latency. This behavior is desirable in our target scenario, where a robot shall react quickly to fast approaching objects suddenly entering the field of view, and attracting its attention. Furthermore, the combination of RFs with different size, processing events on the same field of vision, allows working with a wider operative range of speeds. In the final application, this motion detection module will be used as one of the feature maps used to compute the salience of inputs in the field of view, directing the attention of the robot to potentially relevant stimuli that will be further processed once a saccadic eye motion will place the salient region in the fovea. A strong and low latency response of peripheral sEMDs to fast stimuli could override the salience of static objects. The characterization of the response of the sEMDs in the non-uniform down sampling shows the same qualitative overall behavior for real-world stimuli, showing robustness to noise and to changing the overall spiking activity of the input. The analysis of the individual responses of the sEMDs at different distance from the fovea shows variability that depends on the discretisation of the receptive fields and on the uneven distribution of the receptive field sizes. This effect possibly depends on the Cartesian implementation of the eccentricity, that approximates the distribution of the receptive fields with a rectangular symmetry. A polar implementation of the same concept will reduce the effects of discretisation and improve the overall population response. In a polar implementation, the direction of each sEMD will be aligned along the polar coordinates (radius and tangent), rather than along the Cartesian directions, further improving the variability in the overall response of individual modules and allowing decoding of stimulus direction beyond the cardinal ones.

## Data Availability Statement

The datasets generated for this study can be found in the https://github.com/event-driven-robotics/sEMD-iCub.

## Author Contributions

GD'A: main author of the manuscript and developer of the software. CB, EC, and MM: supervision assistance and review. JO'K and TS: review assistance. EJ: assistance during experiments and writing the manuscript.

## Conflict of Interest

The authors declare that the research was conducted in the absence of any commercial or financial relationships that could be construed as a potential conflict of interest.

## Footnotes

^1^We explored the real-world applicability of the underlying motion detection mechanism prior to this work in which we demonstrated the functionality of the underlying given variable contrast and event-rates in natural environments (Milde et al., 2015, 2018; Schoepe et al., 2019).
