# Supplementary material for: Event-Based Eccentric Motion Detection Exploiting Time Difference Encoding
Source: Front Neurosci. 2020 May 8;14:451. doi: 10.3389/fnins.2020.00451 (PMC7227134; doi:10.3389/fnins.2020.00451)
Supplement: Supplementary file 1 [file Image_1.pdf]

Supplementary Material

1 EXPERIMENTS REAL-WORLD DATA

Final experiments were conducted using recorded data as input to understand the behaviour of the model to a real scenario. We analysed various recordings showing a black bar moving from left to right on a white canvas. The datasets differ in terms of stimulus speed. Given that we want to simulate a real robotic scenario we manually moved the bar in front of the camera. However, as the bar was moved manually, a constant velocity could not be guaranteed. Hence, this is not a comparison with the simulated input experiments because is out of the scope of this analysis. The aim of these recordings was to show the real-world response of the model and its robustness in detecting the correct speed regardless to the noise while decreasing the incoming events from the cameras thanks to the eccentric down-sampling.

2 EXPERIMENTS REAL-WORLD DATA RESULTS

Figure S1 shows the response of the sEMD with eccentric down-sampling for real-world input data from the ATIS camera. Three different stimulus speeds are visualised, ranging from slow S1 a), to medium S1 b) and fast S1 c). As the stimuli were moved manually, no more reliable assertion of the velocities can be made. The observed responses showed the same trend observed for the simulated data. An increase of stimulus speed causes a shift of the area of highest response from the fovea to the periphery. However, unlike observed with the simulated data, the mean firing rate seems to decrease instead of increase for increasing stimulus velocities. A possible explanation for this is the ATIS cameras inherent noise around boarders of the moving bar causing false triggers at slow speeds. Furthermore, as the recordings were performed in an open space with many possible interference sources, thus the datasets are not ideal. However, the center of mass location of the RFs response still provides information about the stimulus velocity. Thus, showing that the model is suitable for a real-world application.

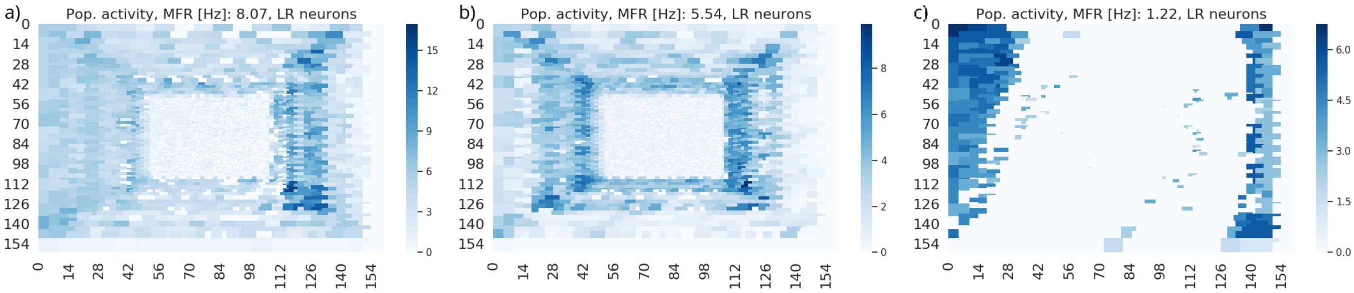

Figure S1. Response from the population of LR sEMDs with the eccentric down-sampling mapped into the cartesian space with a camera resolution of 160x160 pixels. The color-code heatmap represents the MFR of each RF. Population response to real data for three symbolic speeds: slow (a), medium (b) and fast (c).
